# Seed Transcriptome Annotation Reveals Enhanced Expression of Genes Related to ROS Homeostasis and Ethylene Metabolism at Alternating Temperatures in Wild Cardoon

**DOI:** 10.3390/plants9091225

**Published:** 2020-09-18

**Authors:** Hector R. Huarte, Giuseppe. D. Puglia, Andrey D. Prjibelski, Salvatore A. Raccuia

**Affiliations:** 1 CONICET/Faculty of Agricultural Sciences, National University of Lomas de Zamora, 1836 Llavallol, Argentina; hrhuarte@gmail.com; 2 Institute for Agricultural and Forestry Systems in the Mediterranean (ISAFoM), Department of Biology, Agriculture and Food Science (DiSBA), National Research Council (CNR), Via Empedocle, 58, 95128 Catania, Italy; salvatoreantonino.raccuia@cnr.it; 3 Center for Algorithmic Biotechnology, Institute of Translational Biomedicine, St. Petersburg State University, 199004 St. Petersburg, Russia; andrewprzh@gmail.com

**Keywords:** RNA-Seq, dormancy termination, gene expression, antioxidants, ethylene signaling, environmental signals

## Abstract

The association among environmental cues, ethylene response, ABA signaling, and reactive oxygen species (ROS) homeostasis in the process of seed dormancy release is nowadays well-established in many species. Alternating temperatures are recognized as one of the main environmental signals determining dormancy release, but their underlying mechanisms are scarcely known. Dry after-ripened wild cardoon achenes germinated poorly at a constant temperature of 20, 15, or 10 °C, whereas germination was stimulated by 80% at alternating temperatures of 20/10 °C. Using an RNA-Seq approach, we identified 23,640 and annotated 14,078 gene transcripts expressed in dry achenes and achenes exposed to constant or alternating temperatures. Transcriptional patterns identified in dry condition included seed reserve and response to dehydration stress genes (i.e., *HSPs*, peroxidases, and *LEAs*). At a constant temperature, we observed an upregulation of ABA biosynthesis genes (i.e., *NCED9*), ABA-responsive genes (i.e., *ABI5* and *TAP)*, as well as other genes previously related to physiological dormancy and inhibition of germination. However, the alternating temperatures were associated with the upregulation of ethylene metabolism (i.e., *ACO1*, *4,* and *ACS10*) and signaling (i.e., *EXPs*) genes and ROS homeostasis regulators genes (i.e., *RBOH* and *CAT*). Accordingly, the ethylene production was twice as high at alternating than at constant temperatures. The presence in the germination medium of ethylene or ROS synthesis and signaling inhibitors reduced significantly, but not completely, germination at 20/10 °C. Conversely, the presence of methyl viologen and salicylhydroxamic acid (SHAM), a peroxidase inhibitor, partially increased germination at constant temperature. Taken together, the present study provides the first insights into the gene expression patterns and physiological response associated with dormancy release at alternating temperatures in wild cardoon (*Cynara cardunculus* var. *sylvestris*).

## 1. Introduction

Seed dormancy is a continuum process through which dispersed seeds continually sense their surrounding environment perceiving essential information about the most suitable moment to germinate [1,2]. This perception allows modulating seed dormancy level in a cycling way from a high to a low level and vice versa until the suitable germination conditions are met [3]. Environmental temperature, namely constant temperature, acts as a dormancy-alleviation factor, gradually reducing the level of dormancy of the seed population [4]. As the dormancy level is reduced, the ranges of water potential and thermal conditions suitable for germination completion become wider. However, a lot of species still require the presence of some external signals to definitively terminate the dormancy state. Among these, alternating temperatures and light act as dormancy-termination factors removing the ultimate constraint for germination completion once dormancy is sufficiently low [3,4]. Their effect consists of a rapid increase of germination of seeds that have a lowered dormancy degree [5,6]. The daily alternation between low night and high day temperature is an important environmental signal that seeds of some species are adapted to perceive [1,7]. This can provide information on the presence of other plant competitors and the depth of the soil similarly to light [3,8]. This sensing can be very useful, especially for weeds living in variable environments such as the Mediterranean basin [9,10]. Despite the importance of alternating temperatures as a dormancy-termination factor for the completion of germination, little is known about the regulation at the physiological and molecular level of this essential step [11]. Alternating temperatures have recently been found to inhibit abscisic acid (ABA) synthesis through the downregulation of *9-CIS-EPOXYCAROTENOID DIOXYGENASE (NCED*), an enzyme committed to ABA biosynthesis altering the ABA/GA hormone balance [12]. Otherwise, alternating temperatures may act on decreasing ABA sensitivity, as recently postulated for *Polygonum aviculare* [13]. Beyond the GA/ABA hormone ratio, ethylene is actively involved in the promotion of seed germination and acts antagonistically to ABA during *Arabidopsis thaliana* seed development and several other species [14,15,16,17]. Its role in breaking seed dormancy is still not completely ascertained, but there is evidence suggesting that ethylene minimally contributes during dormancy inception, while its major action is during seed imbibition to terminate dormancy and/or initiate germination via crosstalk between ABA and GA pathways [14,18]. This was proposed to determine a decreasing sensitivity to endogenous ABA in concert with GAs to promote these transitional changes leading to germination completion [17]. However, the real magnitude of ethylene contribution to dormancy termination remains to be unveiled. Moreover, there is no evidence of ethylene participation as a part of physiological mechanisms underlying seed exposure to alternating temperatures. Despite reactive oxygen species (ROS) having been considered for a long time as only damaging compounds, in the last decades, they have emerged as key players in seed physiology [19,20]. Recent studies suggest that ROS act as a convergence point of hormonal networks driving cell functioning towards germination through a cross-talk with the major hormonal regulators, i.e., ABA, GA, or ethylene, determining a “ROS wave” [21]. This is carried out by various forms of ROS signaling compounds (e.g., superoxide, hydrogen peroxide and hydroxyl radical) in seeds [19]. In *A. thaliana* the addition in the germination medium of methyl viologen, a ROS-generating compound, partially released seed dormancy, while in sunflower, it alleviated significantly dormancy activating downstream elements of the ethylene signaling pathway but without altering ABA production [20,22,23]. In wild cardoon, previous studies showed an increment of germination in the presence of H_2_O_2_ [24,25]. On the other hand, when ROS level exceeded a certain value, the activation of antioxidant systems was observed in many species, which allows maintaining ROS homeostasis within the oxidative window for germination [26]. To date, transcriptome investigation has been scarcely applied in seed physiology since it was considered to provide only a partial understanding of the cellular events regulating seed dormancy alleviation or termination processes [27,28]. However, many recent contributions showed that, especially for species for which there is a lack of molecular data, transcripts composition analysis provides new insights on gene interactions and their regulatory mechanisms [29,30,31]. Microarray analysis showed that thermal oscillations elicited almost immediate large transcriptome changes in leafy spurge seeds exposed to alternating temperatures [5,32]. Moreover, a mitochondrial matrix-localized heat shock protein, HSP24.7, was shown to represent a critical factor that positively controls seed germination via temperature-dependent ROS generation in cottonseed [33]. However, the molecular dynamics during the dormancy termination step remains largely unknown, especially for non-model organisms that lack genetic and physiological data. The botanical species *Cynara cardunculus* L. includes globe artichoke (subsp. *scolymus* (L.) Hegi), cultivated cardoon (var. *altilis* DC.) also known as industrial cardoon for its bioenergy crop uses [34,35] and the wild cardoon (var. *sylvestris* (Lamk) Fiori) that is considered the progenitor of the globe artichoke [36]. Previous studies investigated the germination physiology of the wild variety, demonstrating that alternating temperatures are useful to terminate achenes dormancy causing an abrupt increase of germination percentage, especially in dry after-ripened achenes [24]. This effect was postulated to be triggered by embryo growth potential with a hormonal regulation through a reduction of gibberellins (GAs) and abscisic acid (ABA) ratio and a decrease in ABA sensitivity [12,37]. To date, limited information has been revealed about the transcriptional regulation in cardoon. The recent publication of a low coverage artichoke genome [38], as well as the investigation of cultivated cardoon flowering transcriptome [39], represent novel essential tools to get further insights about the physiological basis of environmental sensing in wild cardoon. In the present study, we analyzed the transcriptome patterns changes associated with imbibition at alternating temperature using after-ripened achenes with a lowered dormancy level to specifically investigate the dormancy termination process. Since transcriptome dynamics associated with the stimulatory effect on dormancy termination of alternating temperature have not been elucidated for any species, we used a gene co-expression approach analysis to identify gene expression associations that may be involved in the regulation of this process. Moreover, to widen our understanding of the underlying physiological modulation, we investigated the changes in ethylene metabolism in achenes exposed to alternating temperatures and the germination response to compounds able to reduce or increase the ROS content. Altogether our results provide further insights to the dormancy termination process stimulated by alternating temperatures including specific transcriptional patterns and regulation of ROS and ethylene levels.

## 2. Materials and Methods

### 2.1. Achenes Collection

Mature achenes of wild cardoon, *Cynara cardunculus* var. *sylvestris*, were collected from 20 randomly selected plants exhibiting mature capitula (with a fully expanded pappus and easily detachable achenes from the receptacle) growing at a plot in Llavallol, Buenos Aires Province, Argentina (34°27′ S; 58°26′ W) during January of 2019. After collection achenes from different plants were put together and treated as one lot, cleaned and exposed to dehumidification airflow by 24 h to reach a moisture content of approximately 4–5% on a fresh weight basis assessed using humidity measuring instrument (Rotronic, Ettlingen, Germany). The cleaned achenes lot was stored for 7 months at −18 °C (using a freezer) in tightly closed jars filled to 50% with silica gel to maintain the initial moisture level and silica gel was replaced as soon we observed color turning. This was performed to preserve the physiological state of achenes and to prevent ageing. To alleviate achene dormancy and to analyze the effect of different temperature regimes on dormancy termination, for all the tests performed in the present study, we used dry after-ripened achenes at 35 °C for 21 days as reported in greater detail in Huarte et al. [24].

### 2.2. General Procedures for Germination Tests

Dry after-ripened achenes were placed in 9-cm diameter Petri dishes over two pieces of filter paper wetted with 7 mL of distilled water or the corresponding treatment solution. Germination tests (four technical replicates of 25 achenes each) were performed in darkness through wrapping in a double layer of aluminum foil each dish. Darkness was used to prevent the interference of light presence as dormancy termination cue. Achenes were imbibed at 20/10 °C (hereafter referred to as alternating temperatures) with a 12 h thermo-period, or 15 °C (constant temperature) in germination chambers with controlled temperature conditions (±1 °C). Moreover, we also used constant temperatures of 10 and 20 °C to observe wild cardoon germination behavior at the minimum and maximum temperatures of the selected alternating thermal regime. Germination was scored daily, and we keep on monitoring it for 14 days after the last achene germination (unless otherwise stated). Achenes with visible radicle protrusion were considered as germinated and then removed. Data were subjected to ANOVA and means were separated using Tukey’s test at P, 0.05. Data were analyzed using Infostat.

### 2.3. Achenes Treatments and RNA Extraction

To annotate the wild cardoon seed transcriptome and analyze the expression dynamics of selected genes related to seed dormancy termination, we exposed achenes to three different conditions: dry achenes, 48 h imbibed achenes at alternating temperature, 48 h imbibed achenes at a constant temperature following the conditions described in the previous section. Each condition was made up of three biological replicas, and for each replica, we used 30 achenes which were immediately immersed in liquid nitrogen and ground as a whole to a fine powder. Total RNA extraction was carried out starting from about 100 mg of the obtained fine powder using RNAeasy Plant Mini Kit (Qiagen, Hilden), with DNase treatment following the manufacturer’s protocol. RNA quality and quantity were determined using Eppendorf BioSpectrometer (RNA program) and QIAxcel RNA QC Kit (Qiagen, Hilden) selecting only RNA samples with a RIN/RIS/RQN > 7 to be used for downstream analyses, i.e., RNA-Seq and qRT-PCR.

### 2.4. Transcriptome Sequencing, Assembly, and Annotation

In the present study, we carried out an explorative transcriptome analysis aimed at the annotation and identification of relevant transcripts in the seed dormancy termination process in wild cardoon. We used two biological replicates for each treatment condition for RNA-Seq analysis. Library preparation and sequencing were outsourced (Eurofins GmbH, Ebersberg, Germany). For each sample, approximately 1 μg of total RNA was used for library preparation applying a strand-specific cDNA libraries synthesis kit (New England Biolabs, Ipswich, MA, USA). The mRNA was selected with a polyA capturing method, fragmented, ligated with adapters, and amplified. Samples from each library were pooled equimolar and paired-end (PE) sequenced using HiSeq2500 (Illumina Technologies, San Diego, CA, USA) platform with chemistry v4 applying the high-output run mode. Illumina reads were analyzed with the FastQC program, and then quality and adaptors, barcodes, polyA and polyT ends were trimmed using Cutadapt v1.16 with default parameters for paired-end reads and Trimmomatic v0.33 [40] in paired-end mode, setting the minimum length to 50bp. Reads were mapped to v.2 of *C. cardunculus* genome available at (www.artichokegenome.unito.it) with Hisat2 aligner [41]. Gene expression levels were estimated with featureCounts [42] using recently updated cardoon gene annotation [39] and expressed transcripts were carried out for further analysis (FPKM > 2). To functionally annotate the obtained transcripts, we aligned them to the publicly available protein databases including NCBI non-redundant (nr) protein database (downloaded in December 2019), using a local BLASTX analysis with an E value cut-off of 10^−25^ and using InterProScan to infer protein function. The results were used with Blast2Go suite program [43] using default parameters to retrieve Gene Ontology (GO) terms and enzyme codes to visualize specific pathways loaded from Kyoto Encyclopedia of Genes and Genomes (KEGG). The composition of genes was investigated through an enrichment analysis of transcriptome using the Fisher’s Exact test and False Discovery Rate (FDR) considering the transcriptome analyzed in this study as “test-set” and the annotated transcriptome including several phenological stages of *C. cardunculus* obtained in Puglia et al. [39] as “reference-set”. The enriched GO list was, then, analyzed with the AgriGO web application, with Benjamini-Hochberg correction (*p*-value ≤ 0.01) to limit the representation to the most enriched terms. Moreover, to provide a general overview of the contribution of TFs within the seed dormancy termination process, we searched for sequence homologous in the v4.0 Plant Transcription Factor Database (www.planttfdb.cbi.pku.edu.cn; downloaded in December 2019) using local BLASTX (E value cut-off of 10^−25^) and we compared their composition among the treatment conditions.

### 2.5. Differential Gene Expression and Co-Expression Network Analysis

To quantify wild cardoon transcripts expressions, we aligned pre-processed quality-trimmed reads on the reference genome, and we calculated the expression values with the aligned read counts for each transcript. HiSat2 software [41] was used to align the reads on the transcript sequences and HtSeq count [44] was used to evaluate gene expression, in terms of Transcripts per Millions (TPM), from the aligned results. The analysis of differentially expressed genes (DEGs) was carried out with edgeR R package following manual directions for testing multiple conditions. In each analysis, a criterion of |log2(Ratio)| ≥ 2 and an FDR of ≤ 0.01 was used. We run a co-expression analysis on the subset of genes previously identified as differentially expressed using the coseq R package [45] with the K-means approach. The correlation matrix was visualized and analyzed by Cytoscape (version 3.7.2) for co-expression network of genes (http://www.cytoscape.org). To evaluate the transcriptional dynamics of ROS and ethylene pathways across the tested conditions, we selected from DEGs the transcripts with ‘antioxidant activity’, ‘cellular response to stimulus’, ‘response to endogenous stimulus’, ‘response to stress’, ‘seed development’ and ‘signal transduction’ GO terms and we plotted their relative expression as a heatmap. Among this set of transcripts, we selected six genes to be used for real-time PCR analysis for RNA-Seq data validation. For each gene, we differentiated the specific isoform by aligning homologous sequences of *A. thaliana* and wild cardoon using Clustal Omega web server (https://www.ebi.ac.uk/Tools/msa/clustalo/). Identified sequences were used to design specific qRT-PCR primers (Table S1) while as a housekeeping gene we used the actin gene primers already identified in a previous study [39]. Starting from the total RNA extractions, we prepared cDNA libraries using the QuantiTect Kit (Qiagen, Hilden, Germany) and performed real-time PCR reactions on a Rotorgene 6000 cycler (Qiagen, Hilden, Germany) with the QuantiNova SYBR Green Kit (Qiagen, Hilden, Germany). For each treatment condition, we used three biological replicates and three technical replicates of each biological replicates. The fold change in all tissues for each gene was calculated concerning dry achenes condition using the 2^−ΔΔCT^ method. The selected genes set were used to validate the expression profiles of RNA-Seq data through a correlation analysis between their expression profiles measured by qRT-PCR and RNA-Seq was calculated with R software.

### 2.6. Analysis of Ethylene and ROS Regulation at Alternating Temperatures

The effect of ethylene synthesis inhibition on dormancy termination was analyzed by incubating achenes at alternating temperatures in the presence of aminoisobutyric acid (AIB) at doses of 0, 100, 200 and 300 µM (otherwise stated, all chemical compounds were purchased from AG Research, Sigma Argentina) or cobalt chloride (CoCl_2_) (Anedra, Argentina) at doses of 0, 1.25, 2.5 mM. Similarly, the effect of the inhibition of ethylene signaling on dormancy termination was tested incubating achenes at alternating temperatures the presence of 1-methylcyclopropene (1-MCP) (Smartfresh, Argentina) (0, 25, 50 and 100 µM) and AgNO_3_ (Anedra, Argentina) (0, 0.25, 0.5 and 1 mM). On the other hand, to evaluate if the presence of an ethylene releasing compound may increase germination at either fluctuating or constant temperature, achenes were imbibed with 2-chloroethyl-phosphonic acid, i.e., ethephon (Tifon, Gleba SA, La Plata, Argentina) at concentrations of 0, 25, 50 and 100 mM. Moreover, the involvement of ROS compounds in seed dormancy termination was investigated incubating achenes at alternating temperatures in the presence of antioxidants, ROS scavengers, ROS donors and ROS synthesis enzyme inhibitors. We used ascorbic acid and glutathione (GSH) at doses of 0, 10, 20, 40, and 60 mM as antioxidant compounds. While, as a ROS scavenger we used Dimethylthiourea (DMTU) for H_2_O_2_ at a dose of 10 mM. To evaluate NAD(P)H oxidase inhibition (an enzyme related to ROS synthesis) was used Diphenyleneiodonium chloride (DPI) at a dose of 0.1 mM. On the other hand, the effect of methyl viologen (a ROS donor) (0, 0.125, 0.25, 0.5, and 1 mM by 4 h) and Salicylhydroxamic acid (SHAM), a Peroxidase inhibitor (Leymarie et al., 2012) at doses of 0, 2.5, and 5 mM was studied. The effect of SHAM presence was tested at 15 and 20/10 °C.

### 2.7. Ethylene Measurements

To quantify the different ethylene content produced regarding imbibition temperatures, we imbibed dry after-ripened achenes in water (0.6 mL) on two sheets of filter paper inside vial tubes and incubated for 3, 4 or 5 days at alternating or constant temperatures until ethylene measurement was carried out. Vials were sealed with a septum (natural rubber) and then with parafilm to avoid loss of ethylene. Furthermore, we replicated these imbibition conditions with other achenes from the same batch to better monitor germination timing within the vials. Achenes were placed inside vial tubes on two discs of filter paper and moistened with 0.6 mL of distilled water. Vials were sealed in the same way (septum plus parafilm). Ethylene concentrations were determined via gas chromatography (Hewlett Packard 4890, Palo Alto, CA, USA) using a prepacked column (Porapak N 80/100 mesh, length 2 m) and a flame ionization detector. The injector, the column, and the detector had temperatures of 110, 90, and 250 °C, respectively. All replicates were measured independently, and the analysis was conducted from day 3 to day 5 from seed imbibition. Ethylene production was determined by integrating the peaks of ethylene produced multiplied by the flow rate and normalized to the achene dry weight.

## 3. Results and Discussion

### 3.1. Effect of Constant and Alternating Temperatures on Germination

**Figure 1:**
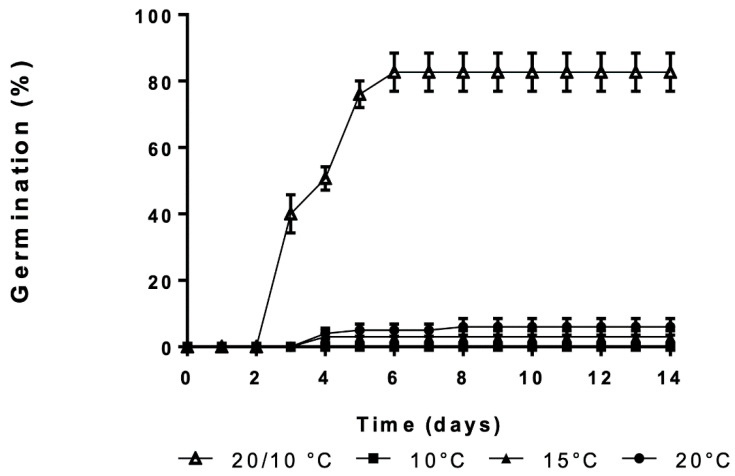
Germination time course percentages of wild cardoon achenes incubated at alternating temperatures (open up-pointing triangle) or constant (closed square, circle, and down-pointing triangle, respectively). Vertical bars indicate the SEs.

Dry after-ripened wild cardoon achenes did not germinate at a constant temperature of 10 °C and only 3 and 6% of achenes germinated at constant 15 and 20 °C (Figure 1). In contrast, the exposure to alternating temperature regimes elicited dormancy termination causing an abrupt increase in germination response up to 80% (*P* < 0.001). The effect of alternating temperatures on germination started from day 3 of imbibition onwards. Maximum germination increased to day 6 and no further germination was scored until the end of the experiment. These results are in line with the previous studies using dry after-ripened wild cardoon achenes, confirming that this treatment can be used for dormancy alleviation in this plant [24,46].

### 3.2. Seed Transcriptome Annotation

In the present study, for the first time for wild cardoon, a seed transcriptome analysis was carried out providing the transcripts composition and dynamics related to the physiological response respect to different imbibition temperatures. We obtained a total of 63,827,612 read pairs with a mean Q always above 34. The datasets generated and analyzed in the current study are available in the NCBI SRA repository PRJNA627453 (https://www.ncbi.nlm.nih.gov/bioproject/ PRJNA627453). Using all the RNA-Seq reads samples, we obtained 20,610 transcripts with an expression value > 0. With the means of the in silico functional annotation, 14,078 genes were identified belonging to 60 GO functional groups including biological process (29 subcategories), cellular component (18 subcategories) and molecular function (13 subcategories) (Figure S1). For biological process, ‘cellular process’ and ‘metabolic process’ were dominant terms, while for molecular function ‘catalytic activity’ and ‘binding’ were the major subcategories. The prominence of ‘binding’ term suggested a crucial role of TFs in seed germination regulation as seen for flower head development in cultivated cardoon [39] or seed transcriptome of other plant families [29]. For cellular components, the identified GO terms were more evenly spread across the subcategories with ‘cell’ and ‘cell part’ accounting for the most numerous ones. Enrichment analysis using the *C. cardunculus* transcriptome [39] as the reference confirmed the relatively higher amount of ‘catalytic activity’ term for molecular function, ‘cell part’ and ‘cell’ for cellular component and ‘metabolic process’ and ‘cellular process’ respect to the *C. cardunculus* transcriptome including several phenological stages (Figure S2). As for biological processes the over-representation of ‘metabolic process’ and ‘cellular process’ reveal the upregulation of pathways, including anabolism and catabolism, and communication occurring among cells. Interestingly, we found the ‘signaling’ and ‘response to stimulus’ terms enriched respect to the reference annotation. Another highly represented functional group was ‘binding’, which includes transcription factors activity important for seed germination. We identified transcripts, 9226 (39.0% of the total number of transcripts) accounting for 57 TF families (Figure S3). In general, the alternating temperature treatment condition exhibited the highest number of genes attributable to TF families, while the dry achenes the lowest. Considering the seed transcriptome including all the treatment conditions, the TF family with the highest number of representatives were bHLH (1026), then MYB/MYB-related (931), NAC (593) and ERF (532), which altogether represent the 33.4% of the identified TFs (Figure S3). Similar TFs families composition was observed in other species [29,30], but in the present study, we observed some TFs families usually not highly represented as GAI-RGA-SCARECROW (GRAS), FRS (FAR1 Related Sequences) and Golden2-like (G2-like) that accounted for the 8.8% of the total identified TFs.

### 3.3. Differential Expression Analysis

**Figure 2:**
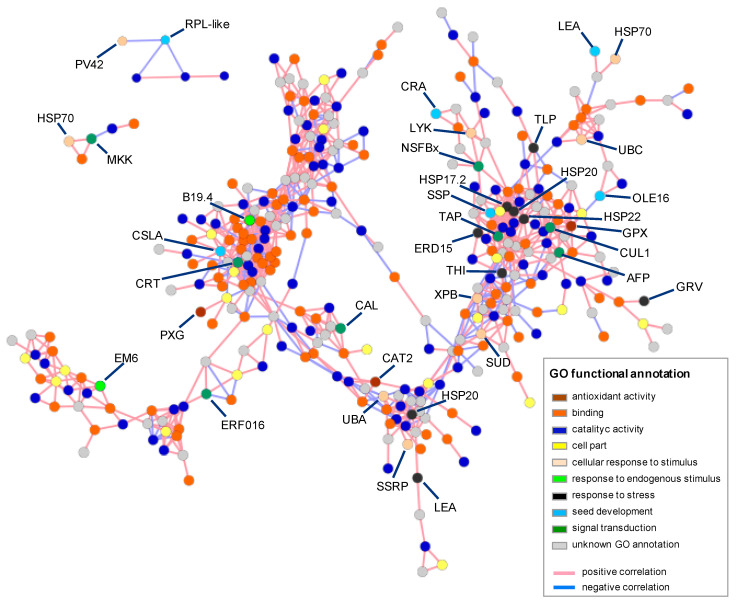
The layout of the three largest connected components of the correlation-based co-expression network obtained using differentially expressed genes (DEGs), in which are reported the putative gene names for antioxidant activity, cellular response to stimulus, response to endogenous stimulus, response to stress, seed development, and signal transduction GO terms. Other independent smaller-size subnetworks are shown in Figure S8.

The analysis of the differentially expressed genes (DEGs) across all the samples resulted in 4737 sequences (Table S2) and their expression profiles were confirmed by qRT-PCR analysis, which showed a good correlation (R^2^ = 0.64) with RNA-Seq data (Figure S4A,B), supporting the reliability of our dataset. The variance of expression levels across the samples confirmed the marked difference of transcriptional regulation produced by the exposition to different environmental factors such as imbibition to constant or alternating temperature (Figure S5). Enrichment analysis on DEGs showed an upregulation of functional GO terms associated with binding and catalytic processes (Figure S6). This expression pattern testifies of an intense enzymatic activity that is supported by the TFs. Similar diversity in transcriptional profile was already reported for *Paris polyphylla* seeds exposed to warm stratification respect to non-stratified seeds [30], but, to the best of our knowledge, this is the first report investigating the transcriptional profile variation during dormancy termination. To identify the most relevant functional groups involved in its regulation, we generated an expressed transcripts matrix that comprised 764 correlated DEGs, which consisted in 131 GO terms, mostly enriched for ‘metabolic process’, ‘cellular process’, ‘biological regulation’ and ‘response to stimulus’ amongst the ‘biological process’ GO category, while ‘binding’, ‘activity of structural molecules’, ‘catalytic activity’ and ‘antioxidant activity’ for ‘molecular function’ GO category (Figure S7 and Table S3). To analyze the association amongst the correlated transcripts, we generated a correlation-based network, which showed the 764 transcripts, as nodes, connected by 1344 edges, at Pearson correlation coefficient of 0.90 (Figure S8). The largest connected component of the network with 347 nodes and 943 edges is shown in Figure 2, which functional annotation is reported in Table 1 for selected genes which GO functional annotation was associated with seed dormancy, ethylene, and ROS homeostasis.

Binding and catalytic activity GO terms were uniformly spread across the network. On the contrary, some putative transcripts encoding for response to ROS stress as *GLUTATHIONE PEROXIDASE (GPX*), reported being upregulated in the presence of oxidative stress [47], or as *HEAT-SHOCK PROTEINS (HSPs*) [63] were not uniformly distributed across the network, and formed a closely associated cluster. Within this group, we observed the presence of a cullin protein (*CUL1*), the family of which is associated with degradation of *ABA INSENSITIVE 5 (ABI5*) [50] and *ABI FIVE BINDING PROTEIN (AFP*) that participates in the control of *ABI5* accumulation [55]. This tight association can suggest a close interaction of these transcripts in the removal of the last dormancy constraints. On the other hand, this group also included *TYPE 2A PHOSPHATASE-ASSOCIATED PROTEIN 46 (TAP46)*, which is known to stabilize *ABI5* transcript expression [54]. Therefore, the nature of their interaction should be further investigated to unveil how they can modulate ABA levels. Another smaller cluster consisted of cardoon homologous to *PV42* (an *SNF1*-related protein kinase regulatory subunit gamma-like), a *RIBOSOMAL PROTEIN L-LIKE (RPL-LIKE),* and three catalytic genes. *SNF1* and *RPL* interaction was associated with the completion of germination in Arabidopsis seeds [64]. Moreover, *MITOGEN-ACTIVATED PROTEIN KINASE 9-like (MKK)*, which is associated with the induction of ethylene synthesis [53], was not included in the main network and the annotation of closely linked genes is not sufficiently clear for this plant species to speculate for a possible interaction among them. Similarly, other cardoon putative transcripts associated with ethylene such as *RESPONSIVE TRANSCRIPTION FACTORS (ERF*), 1-*AMINOCYCLOPROPANE-1-CARBOXYLIC ACID OXIDASE (ACO), AMINOCYCLOPROPANE-1-CARBOXYLIC ACID SYNTHASE (ACS*) or with the modulation of ROS, such as *RESPIRATORY BURST OXIDASE PROTEIN* (*RBOH*) were not included in the main subnetwork.

**Figure 3:**
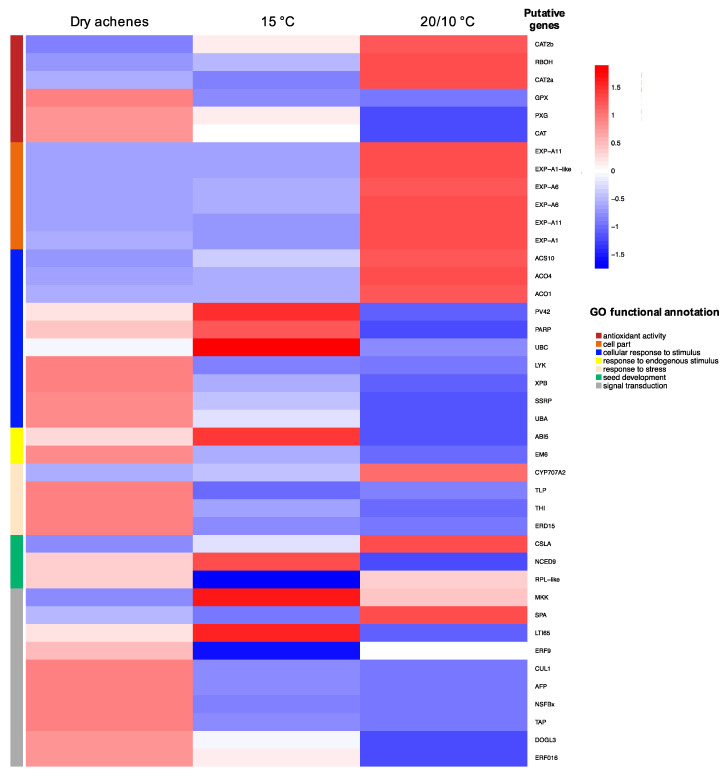
Expression heatmap of a set of transcripts associated with seed dormancy, ethylene, and ROS homeostasis in dry achenes, imbibed achenes at constant temperature (15 °C) and imbibed achenes at alternating temperatures (20/10 °C). The color scale represents the log2-transformed TPM value.

However, when we analyzed the transcriptional dynamics of a broader set of genes (Figure 3), we observed an increase of dehydration stress response as *HSPs*, *PEROXIDASE* (*GPX*), *PEROXYGENASE* (*PXG*), and *LATE EMBRYOGENESIS ABUNDANT* (*LEA*) in dry achenes. Moreover, in this condition, there were a richer composition of *SEED STORAGE PROTEINS* (*SSP*), Oleosin proteins and DNA repair system factors, such as *XPB*. These findings are in line with transcripts composition recently described for dry *A. thaliana* seeds [64]. However, at a constant temperature, the transcriptional program changed abruptly with the expression of homologous genes associated with ABA signaling and biosynthesis, as can be drawn from the upregulation of *ABI5, TAP, PV42* and *LTI65* transcripts expression. These results suggest increased ABA biosynthesis at a constant temperature, which is supported by the upregulation of *NCED9* at this condition. Respect to dry seed, at the constant temperature we observed a downregulation of *DELAY OF DORMANCY-LIKE3 (DOGL3)*. This expression is further dramatically reduced at alternating temperatures. Recently, Sall et al. [64] reported that the overexpression of *DOGL3* caused ABA hypersensitivity in seed germination of *A. thaliana* but proposed a role as an inducer of seed reserve accumulation for *DOGL* genes respect to dormancy modulator which characterizes *DOG1*. Further research is needed to confirm whether *de novo* ABA biosynthesis associated with exposure to constant temperature is the main mechanism involved in the dormancy maintenance of wild cardoon. The alternating temperature condition, instead, stimulated the expression of *RBOH* that is responsible for the biosynthesis of superoxide and was associated with dormancy alleviation in sunflower [20]. The differential expression of some *CATALASE* family genes can support the presence of an oxidation stress control system acting differently depending on the physiological step. *CYP707A2* associated with ABA degradation [51] and *RPL* reported as a stimulator of germination completion [64]. Moreover, transcripts encoding for ethylene metabolism were upregulated, such as *ACO1*, *ACO4,* and *ACS10,* and also signaling with *EXPANSINs*. The latter is responsible for plant cell wall loosening through ethylene promotion of micropylar endosperm weakening by inducing the expression of *CELL WALL REMODELLING PROTEINS* (*CWRP*) and/or ROS that cause cell wall loosening or cell separation of this tissue [65]. The upregulation of *CELLULOSE SYNTHASE-LIKE A* (*CSLA*) at the same imbibition condition can probably be reconducted to this reorganization of plant cell walls for germination completion. Whether the stimulation of ROS signaling and ethylene biosynthesis and signaling has a major role in dormancy termination of wild cardoon needs to be confirmed by further studies unveiling the interaction among their pathways.

### 3.4. Effect of Incubation Temperature on Ethylene Synthesis and Germination

**Figure 4:**
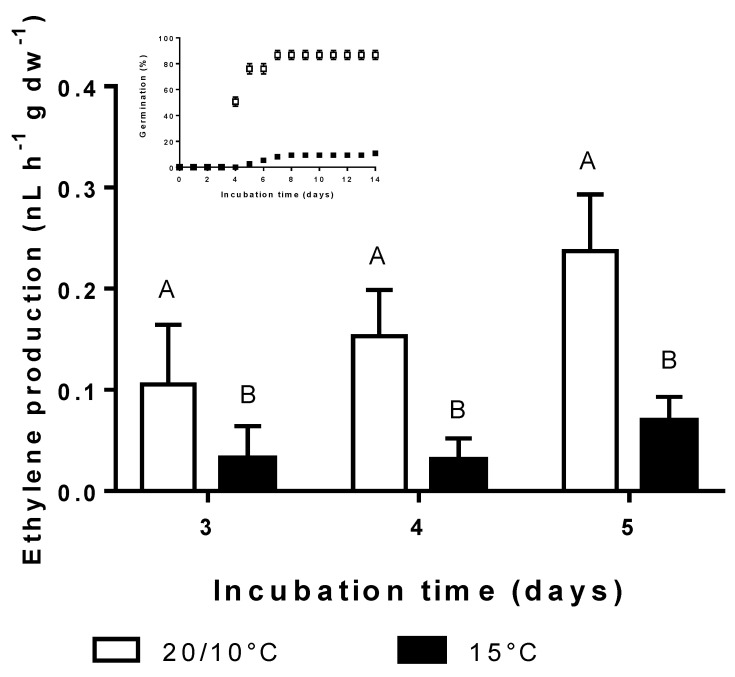
Ethylene production (±SE) in achenes exposed to constant 15 °C (closed bars) or alternating temperatures 20/10 °C (open bars) for 5 days.

Ethylene production was detected one day before the beginning of germination and its content was always significantly higher at alternating temperatures in comparison with that scored at constant temperature (Figure 4). Ethylene content at day 3 was 0.31 and 0.06 for 20/10 °C and 15 °C, respectively. It gradually increased at alternating temperatures to reach its maximal value at day 5 of incubation (0.78 ppm). In contrast, at the same time, just 0.23 ppm was measured at constant temperatures. Similar results on ethylene production during seed germination were previously published [17].

**Figure 5:**
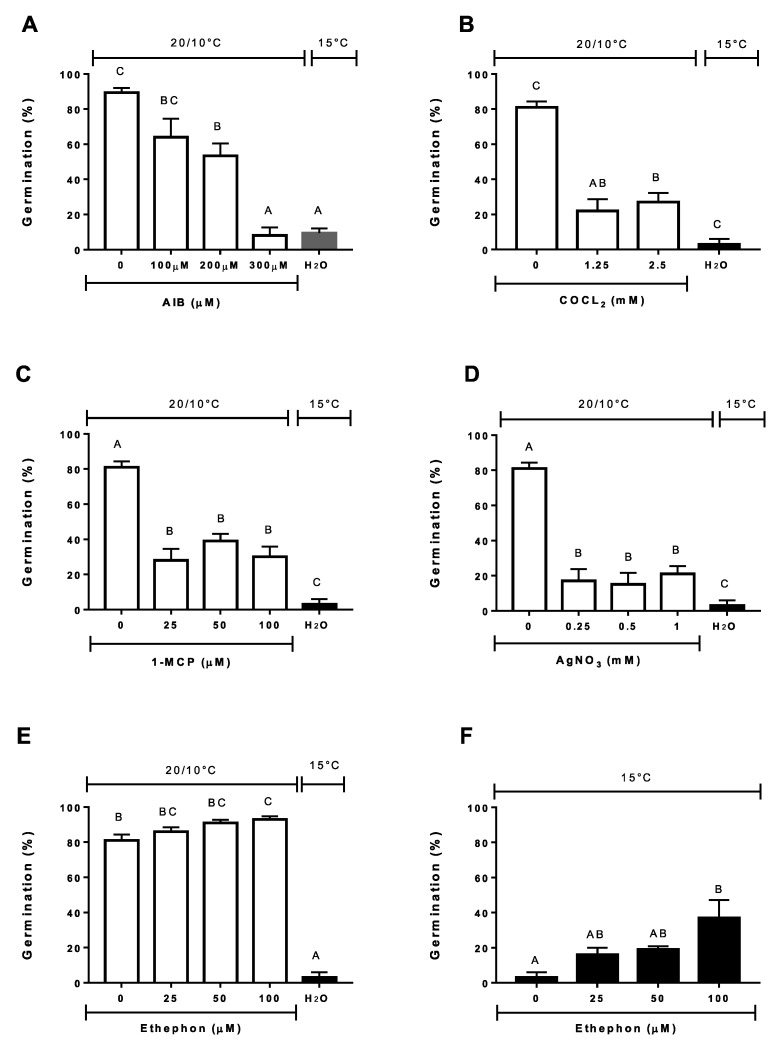
Final germination percentages of wild cardoon achenes incubated at alternating temperatures (20/10 °C) (open bar) or constant (15 °C) in the presence of ethylene biosynthesis inhibitors (**A**,**B**), inhibitors of ethylene binding to its receptor (**C**,**D**), or ethylene donor (**E**,**F**). Vertical bars indicate the SEs. Similar letters at the top of each bar indicate no differences according to Tukey’s test (*P* < 0.05).

Moreover, the important role of ethylene in germination completion was also supported by the results obtained from the use of inhibitors of its biosynthesis and signaling. In all cases, germination at alternating temperatures was gradually reduced (Figure 5A–D). For instance, the presence of AIB, an inhibitor of ACC oxidase, reduced germination at alternating temperatures to 8 % being statistically comparable to germination scored at constant temperature of the control (Figure 5A) (*P* < 0.05). Similarly, CoCL_2_ at a dose of 1.25 and 2.5 mM lowered significantly total germination at alternating temperature, compared to the constant temperature of the control (Figure 5B). Also, the interference on ethylene receptors had a significant negative effect on dormancy termination by alternating temperatures (Figure 5C,D). The use of 0.25–1 mM AgNO_3_ and 25–100 µm 1-MCP inhibit the germination up to 50%, respectively. On the other hand, the germination was only partially increased in the presence of ethephon, an ethylene releasing compound, (Figure 5E,F). At alternating temperature, ethephon increased germination to 91%, but at constant temperatures, the germination response was lower than 40%. That is, the ethylene alone did not replace the requirement of alternating temperatures for dormancy termination. Germinations scored at constant temperatures by the use of ethephon agree with that reported by Kepczynski and Sznigir [66] using 16 weeks dry after-ripened *Amaranthus retroflexus* seeds and similar to Corbineau et al. [18]. Ethephon replaces the requirement of dormancy alleviation factors, such as cold stratification and dry after ripening, in several species presenting primary dormancy [18]. Thus, these results led us to suggest that alternating temperatures elicits ethylene biosynthesis and probably signaling as well. However, the increase of ethylene production does not necessarily imply that its presence can determine dormancy termination in wild cardoon. This hormone plays an important role in the modulation of last dormancy constraint in concert with other key players, such as ABA/GA and/or ROS [18]. Further research is needed to ascertain whether this is the major mechanism for ABA/GA balance modulation able to produce a reduced ABA sensitivity, as previously described in the presence of alternating temperatures [12,37].

### 3.5. Effect of Antioxidants, ROS Scavengers, and ROS Donors on Germination at Alternating or Constant Temperatures

**Figure 6:**
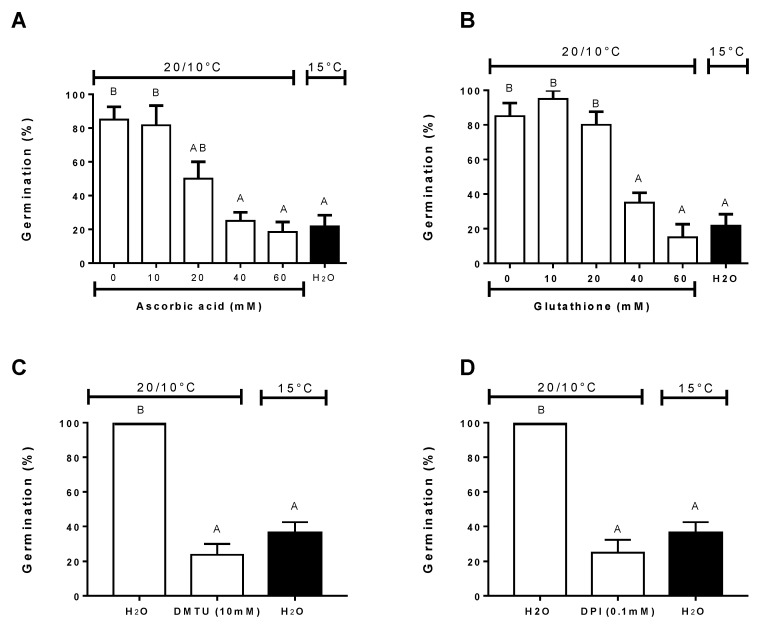
Final germination percentages of wild cardoon achenes incubated at alternating temperatures (20/10 °C) (open bar) or constant (15 °C) in the presence of antioxidant compounds (**A**,**B**), hydrogen peroxide scavenger (**C**), and NAD(P)H oxidase inhibitors (**D**). Vertical bars indicate the SEs. Similar letters at the top of each bar indicate no differences according to Tukey’s test (*P* < 0.05).

The presence of antioxidants, such as ascorbic acid and glutathione, progressively reduced significant germination in achenes exposed to alternating temperatures determining similar percentages observed at constant temperature of the control (Figure 6A,B). This suggests that dormancy termination by alternating temperatures could require the presence of ROS compounds. To support this hypothesis, further germination experiments were carried out in the presence of DMTU, a hydrogen peroxide scavenger, and DPI, a ROS synthesis inhibitor. Both compounds reduced germination to an extent similar to those obtained at constant temperature of the control (*P* < 0.001) (Figure 6C,D). Results obtained for DPI and DMTU are in agreement with those published by Leymarie et al. [20] using *A. thaliana* seeds.

**Figure 7:**
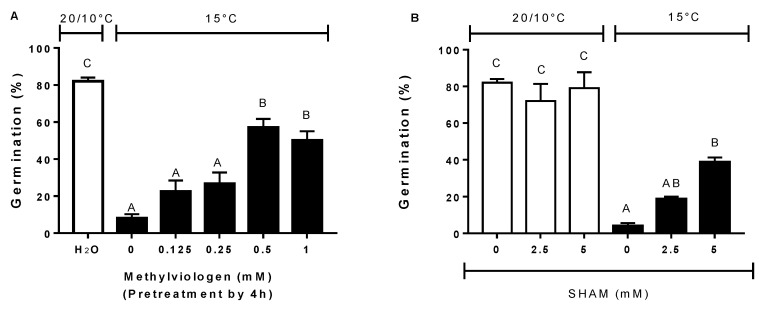
Final germination percentages of wild cardoon achenes incubated at alternating temperatures (20/10 °C) (open bar) or constant (15 °C) in the presence of methyl viologen (**A**) and salicylhydroxamic acid (SHAM) (**B**). Vertical bars indicate the SEs. Similar letters at the top of each bar indicate no differences according to Tukey’s test (*P* < 0.05).

On the other hand, at constant temperature, the use of methyl viologen, a ROS donor, significantly increased germination from 8 to 57% at doses of 0 and 0.5 mM, respectively (Figure 7A), similarly to previous investigations in sunflower achenes [23,67]. Likewise, the inhibition of peroxidase, an enzyme that reduces the amount of hydrogen peroxide, in the presence of SHAM, partially increased germination at constant a temperature (Figure 7B). Although the results obtained in the present study have to be confirmed by direct ROS measurements, altogether these findings suggest that dormancy termination by alternating temperatures may include the involvement of ROS compounds

## 4. Conclusions

The present study is the first large-scale gene expression investigation on dormancy termination process in wild cardoon. Transcriptome patterns associated with the imbibition at constant temperature include upregulation of ABA biosynthesis genes, ABA-responsive genes, as well as other genes previously related to physiological dormancy and inhibition of germination. While expression patterns stimulated at alternating temperatures comprised ethylene and ROS signaling and metabolism together with ABA degradation and cell wall loosening. Physiological assays support molecular data showing that ethylene is necessary for dormancy termination at alternating temperatures, even if its presence does not imply the completion of germination. Similarly, ROS is needed for dormancy termination since its depletion hampers this process, but ROS donors cannot overcome dormancy completely. These findings suggest an important role of both ethylene and ROS in dormancy termination at alternating temperatures, most probably as a fine-tuned mechanism for environmental sensing. This can be a very useful system for effectively achieving dormancy termination once environmental conditions are suitable for germination in highly disturbed habitat in which this plant lives. Moreover, our results may have applications in naturalization efforts using wild cardoon for the naturalization of highly disturbed habitats impacted by human activity (e.g., sowing seeds at the correct environmental temperature regimes).

## Figures and Tables

**Table 1:** Gene annotation of transcripts associated with seed dormancy, ethylene, and reactive oxygen species (ROS) homeostasis.

Gene Identifier	Gene Description	Effect on Dormancy Regulation
Ccrd_v2_22156_g15	*GPX—phospholipid hydroperoxide glutathione peroxidase*	Reduction of H_2_O_2_ or organic hydroperoxides [47]
Ccrd_v2_22208_g15	*RBOH—Respiratory burst oxidase protein*	Biosynthesis of superoxide/Dormancy alleviation factor [20]
split_gene_Ccrd_v2_02613_g01-g146	*CAT2—Catalase-like isoform X2*	Protect from H_2_O_2_ and lipid peroxidation [48]
Ccrd_v2_14857_g10	*PXG—Plant seed peroxygenase*	Protect from dehydration stress [49]
Ccrd_v2_04883_g02	*DOGL3—protein DOG1-like 3 isoform*	Effect unclear
Ccrd_v2_02779_g02	*CUL1—Cullin-1-like isoform X1*	Control of ABA biosynthesis [50]
Ccrd_v2_12828_g08	*ERF9—ETHYLENE RESPONSE FACTOR9*	Ethylene signaling [51]
Ccrd_v2_23452_g16	*SPA—protein SUPPRESSOR OF PHYA-105 1-like isoform*	Regulates circadian rhythms/germination enhancer [52]
Ccrd_v2_21316_g15	*MKK—Mitogen-activated protein kinase 9-like*	Induces the synthesis of ethylene [53]
Ccrd_v2_08680_g05	*NSFBx—Probable F-box protein (At5g04010)*	Effect unknown
Ccrd_v2_00002_g01	*TAP—2A phosphatase associated protein*	Dormancy enhancer [54]
Ccrd_v2_09661_g05	*AFP—ninja-family protein AFP3-like*	Control of ABA biosynthesis [55]
Ccrd_v2_03046_g02	*ERF016—Ethylene-responsive transcription factor ERF016-like*	Effect unclear
novel_gene_1_5b8548b9	*LTI65—low-temperature-induced 65 kDa protein-like*	Responsive to ABA [56]
Ccrd_v2_06887_g03	*PV42—SNF1-related protein kinase regulatory subunit gamma-like PV42a*	Dormancy enhancer [57]
Ccrd_v2_15915_g11	*PARP—putative Poly [ADP-ribose] polymerase 3*	DNA protection system
Ccrd_v2_01121_g01	*UBC—Ubiquitin-conjugating enzyme E2 2*	Induced by ABA [58]
Ccrd_v2_24782_g17	*ACO1—1-AMINOCYCLOPROPANE-1-CARBOXYLATE OXIDASE1*	Ethylene biosynthesis [51]
Ccrd_v2_23833_g17	*ACO4—1-AMINOCYCLOPROPANE-1-CARBOXYLATE OXIDASE4*	Ethylene biosynthesis [51]
Ccrd_v2_16461_g11	*ACS10—ACC synthase10*	Ethylene biosynthesis [51]
Ccrd_v2_19164_g13	*LYK—LYSIN MOTIF RECEPTOR KINASE*	Effect unknown
Ccrd_v2_01115_g01	*SSRP—FACT complex subunit SSRP1-like isoform X1*	Dormancy enhancer [59]
Ccrd_v2_14002_g09	*UBA—ubiquitin-activating enzyme E1 1-like isoform X*	Effect unknown
Ccrd_v2_22183_g15	*XPB—general transcription and DNA repair factor IIH helicase subunit XPB1*	DNA repair [60]
Ccrd_v2_00258_g01	*ERD15—protein EARLY RESPONSIVE TO DEHYDRATION 15-like*	Induced by dehydration stress/Modulates ABA response [61]
Ccrd_v2_22449_g15	*CYP707A2—Cytochrome P450, Family 707, Subfamily A, Polypeptide2*	Reduced dormancy [51]
Ccrd_v2_15609_g11	*NCED9—NINE-cis-EPOXYCAROTENOID DIOXYGENASE -9*	Dormancy enhancer [51]
Ccrd_v2_13522_g09	*ABI5—protein ABSCISIC ACID-INSENSITIVE 5*	Dormancy enhancer [55]
Ccrd_v2_00305_g01	*EM6—em-like protein GEA6*	Effect unclear
Ccrd_v2_02516_g01	*TLP—thaumatin-like protein 1b*	Effect unknown
Ccrd_v2_05395_g03	*THI—Gamma thionin*	Effect unknown
Ccrd_v2_00955_g01	*CSLA—CELLULOSE SYNTHASE-LIKE (CSL)*	Effect unknown
Ccrd_v2_08837_g05	*RPL-like—Ribosomal Protein-like*	Reduced dormancy [62]
Ccrd_v2_19371_g13	*EXPA6—Expansin A6*	Cell wall loosening/Ethylene signaling [51]
Ccrd_v2_05454_g03	*EXPA11—Expansin A11*	Cell wall loosening/Ethylene signaling [51]
Ccrd_v2_19070_g13	*EXPA11—Expansin A11*	Cell wall loosening/Ethylene signaling [51]
Ccrd_v2_21520_g15	*EXPA1—Expansin A1*	Cell wall loosening/Ethylene signaling [51]
Ccrd_v2_01833_g01	*EXPA1-like—Expansin A11-like*	Cell wall loosening/Ethylene signaling [51]
Ccrd_v2_00414_g01	*EXPA6—Expansin A6*	Cell wall loosening/Ethylene signaling [51]

## Supplementary Materials

The following are available online at https://www.mdpi.com/2223-7747/9/9/1225/s1, Table S1. qRT-PCR primers designed on selected transcripts identified within the transcriptome assembly and used for RNA-seq data validation. Table S2. Expression levels, reported as log2-transformed TPM value, of 4737 DEGs identified in this study. Table S3. Complete list of correlated transcripts present in the co-expression network analysis. Figure S1. In silico functional annotation of identified transcripts. Gene Ontology (GO) terms are reported for each GO category. Figure S2. Enrichment analysis of seed transcriptome annotation compared to the *C. cardunculus* transcriptome including several phenological stages. Figure S3. Amount of transcription factor families identified in this study. Figure S4. qRT-PCR analysis for RNA-Seq data validation. A: Relative expression of wild cardoon homologous transcripts associated with regulation of ethylene, ROS and ABA. The relative expression ratio is expressed as the fold increase relative to dry achenes. The error bars represent the standard error of the mean of three biological replicates. Letters indicate significantly different values according to ANOVA (*p*-value ≤ 0.05). B: Correlation of gene expression results obtained from real-time PCR analysis and RNA-Seq (TPM) for 6 selected genes across samples. The correlation of determination (R2) was 0.64. Figure S5. Hierarchical cluster analysis of differentially expressed genes in dry achenes, imbibed achenes at a constant temperature (15 °C) and imbibed achenes at alternating temperatures (20/10 °C). The color scale represents the log2-transformed TPM value. Figure S6. Enrichment analysis of the 764 highly correlated DEGs. Figure S7. Expression heatmap of the 764 highly correlated DEGs. DS: dry achenes; IDS: imbibed achenes at constant temperature (15 °C); INDS: imbibed achenes at alternating temperatures (20/10 °C). The color scale represents the log2-transformed TPM value. Figure S8. The layout of all the correlation-based co-expression networks obtained using of the 764 highly correlated DEGs, in which are functional GO categories.
