# Supplementary material for: Seed Transcriptome Annotation Reveals Enhanced Expression of Genes Related to ROS Homeostasis and Ethylene Metabolism at Alternating Temperatures in Wild Cardoon
Source: Plants (Basel). 2020 Sep 18;9(9):1225. doi: 10.3390/plants9091225 (PMC7570316; doi:10.3390/plants9091225)
Supplement: Supplementary file 1 [file plants-09-01225-s001.zip › supplementary data/Table S1.docx]

**Table S1.** qRT-PCR primers designed on selected transcripts identified within the transcriptome assembly and used for RNA-seq data validation.

| Gene name | Primer sequence (5’-3’) |
| --- | --- |
| ACO1 | F: GAGCTTCGCAAAGCAATG |
|  | R: GCCTCCTTGATGTATTCCTTA |
| CYP707A2 | F: AGCTGTCACTGATGAACAA |
|  | R: GATAGAAGCAACTCTGAGGG |
| NCED | F: GATTGGCGAGTGTTTTACG |
|  | R: CAAATCATCCTCCGACATC |
| ERS1 | F: TTCTTCCTCGAAGCTTTCTG |
|  | R: CAGATATATACTGATACTTGACCA |
| RBOH | F: CACAATGCATAGGAATGAG |
|  | R: GAGTCCAATACTCGTATAATTCA |
| CAT2 | F: TACACCAGGGAGGGAAACT |
|  | R: TTCTTGGATGTGTGACTTGG |
