# Supplementary material for: Seed Transcriptome Annotation Reveals Enhanced Expression of Genes Related to ROS Homeostasis and Ethylene Metabolism at Alternating Temperatures in Wild Cardoon
Source: Plants (Basel). 2020 Sep 18;9(9):1225. doi: 10.3390/plants9091225 (PMC7570316; doi:10.3390/plants9091225)
Supplement: Supplementary file 1 [file plants-09-01225-s001.zip › supplementary data/Fig S8.pdf]

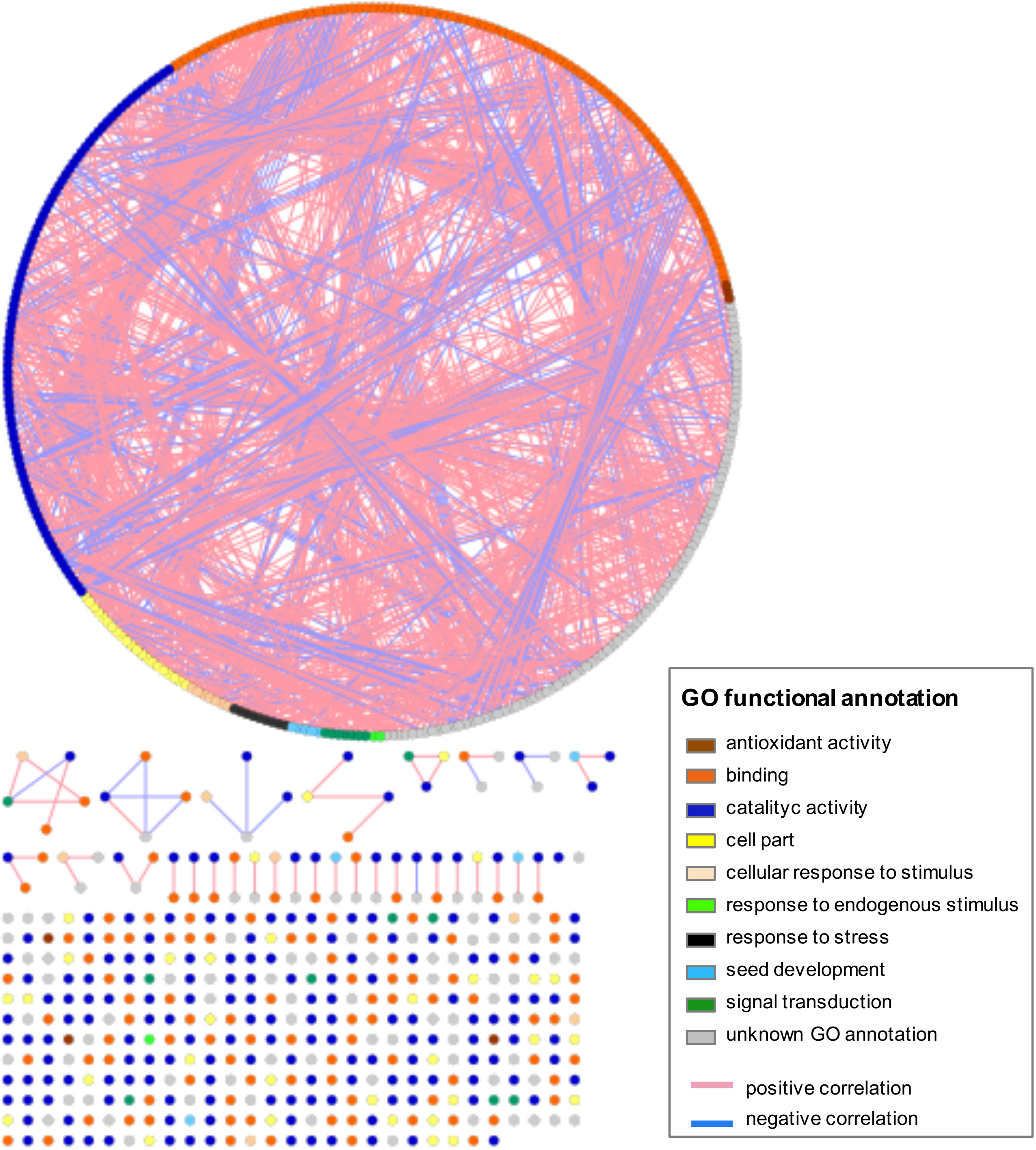
GO functional annotation
antioxidant activity
binding
catalityc activity
cell part
cellular response to stimulus
response to endogenous stimulus
response to stress
seed development
signal transduction
unknown GO annotation
positive correlation
negative correlation
