# Supplementary figures and images for: Seed Transcriptome Annotation Reveals Enhanced Expression of Genes Related to ROS Homeostasis and Ethylene Metabolism at Alternating Temperatures in Wild Cardoon
Source: Plants (Basel). 2020 Sep 18;9(9):1225. doi: 10.3390/plants9091225 (PMC7570316; doi:10.3390/plants9091225)

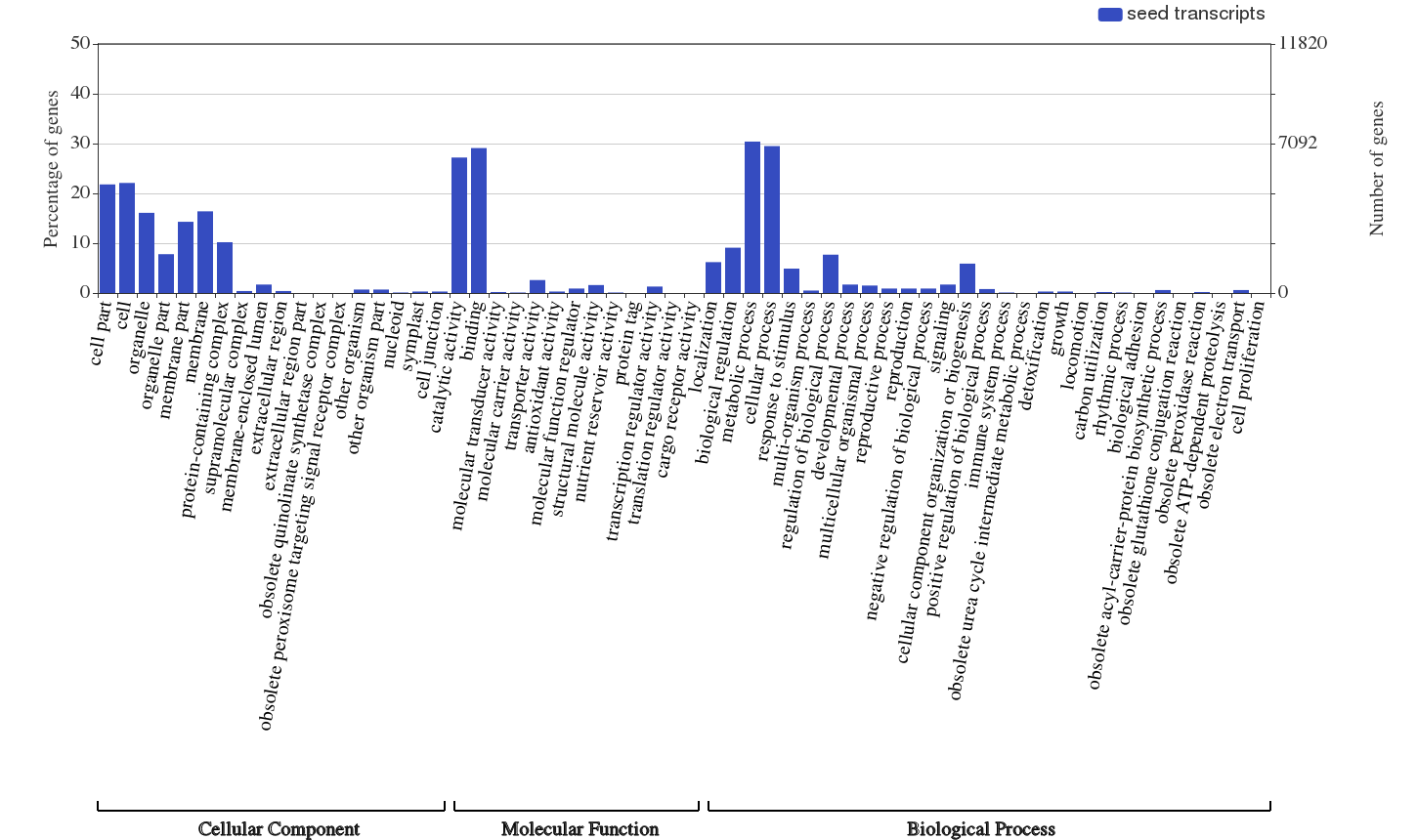

Supplement: Supplementary file 1 [file plants-09-01225-s001.zip › supplementary data/Fig S1.png]

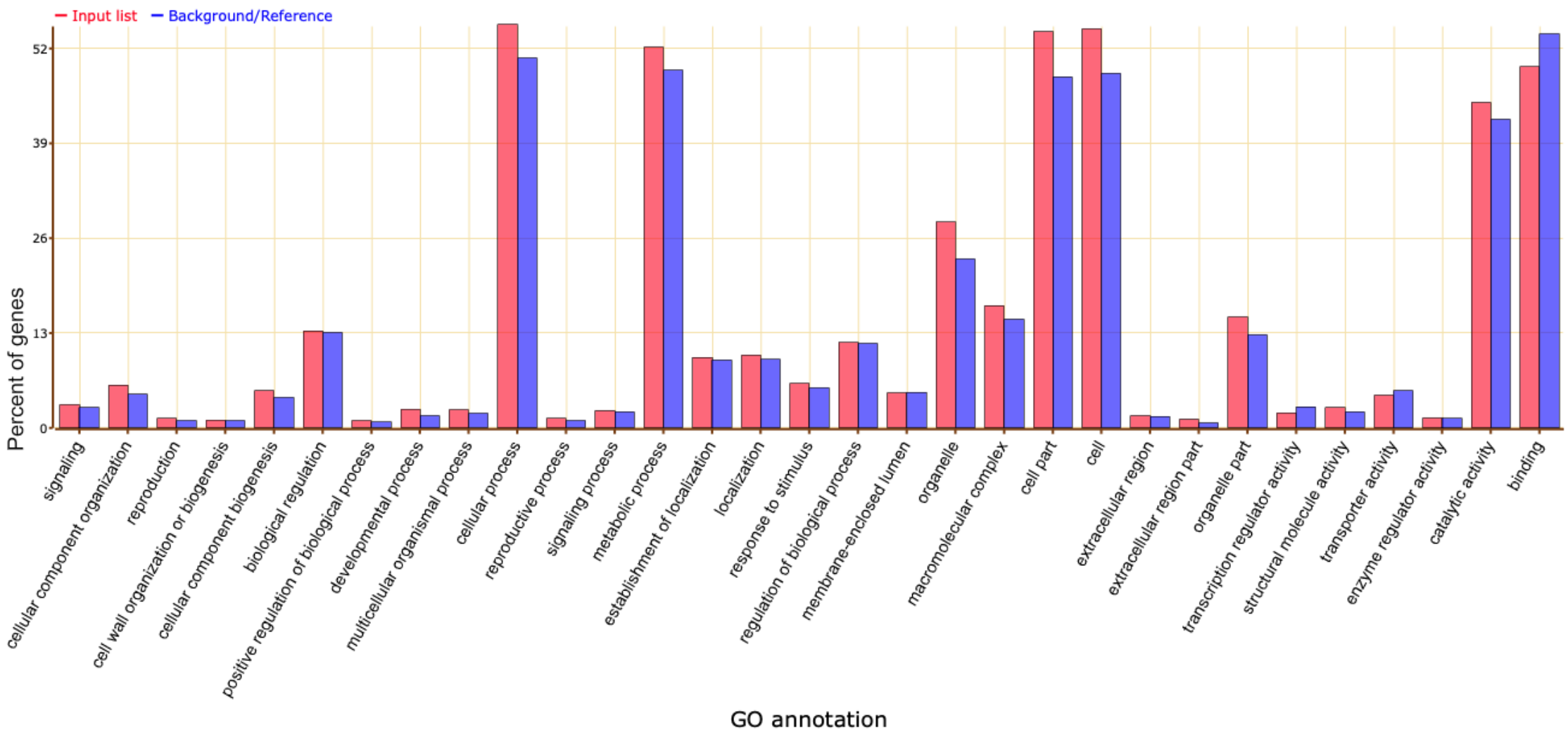

Supplement: Supplementary file 1 [file plants-09-01225-s001.zip › supplementary data/Fig S2.pdf]

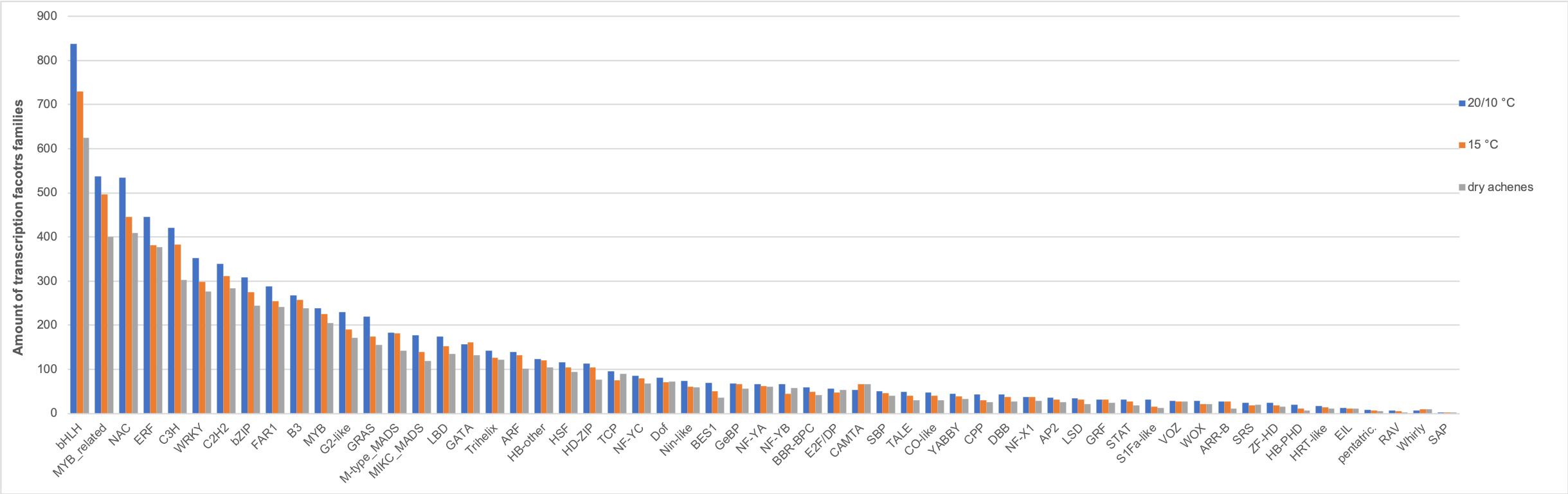

Supplement: Supplementary file 1 [file plants-09-01225-s001.zip › supplementary data/Fig S3.png]

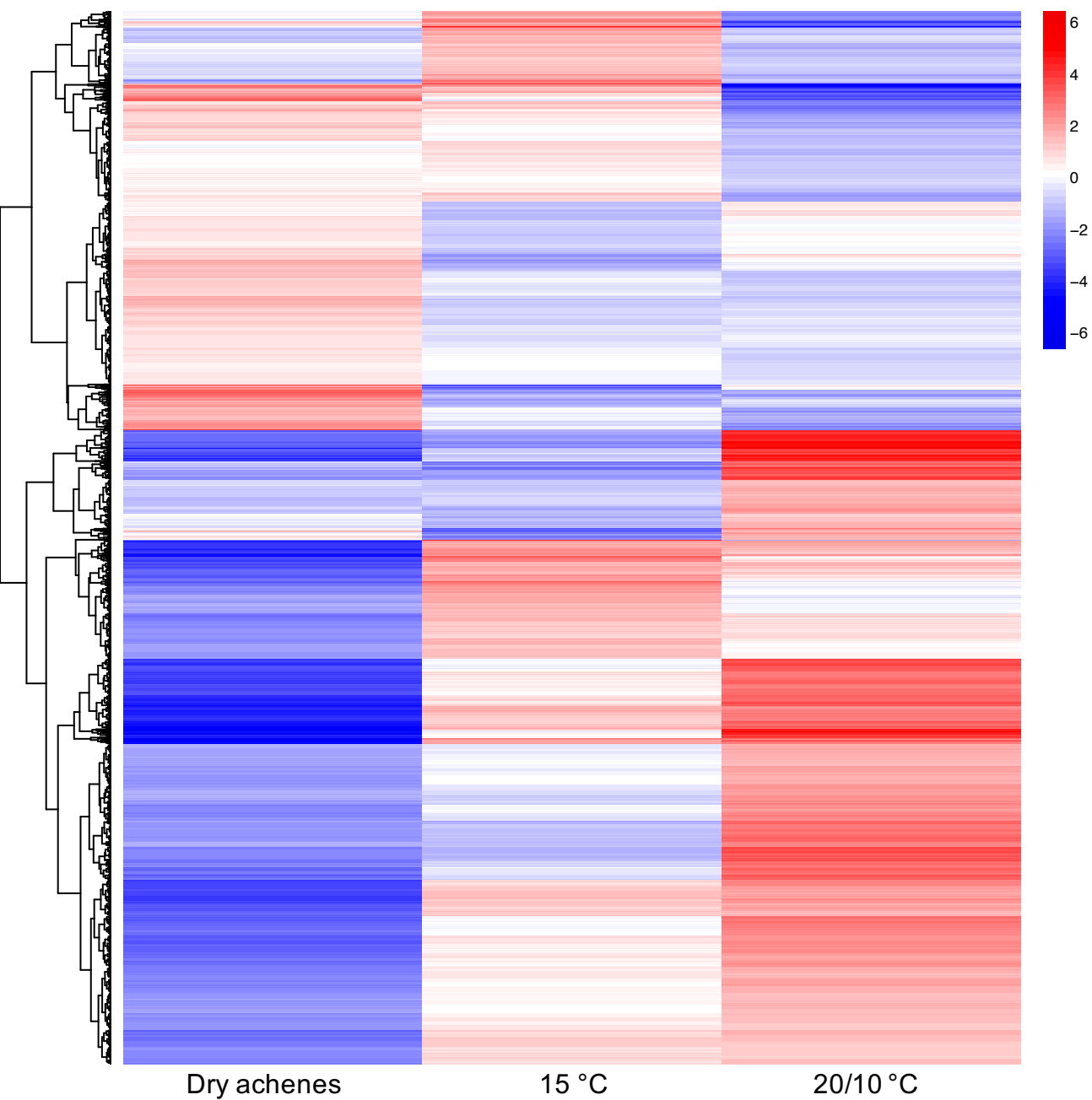

Supplement: Supplementary file 1 [file plants-09-01225-s001.zip › supplementary data/Fig S5.pdf]

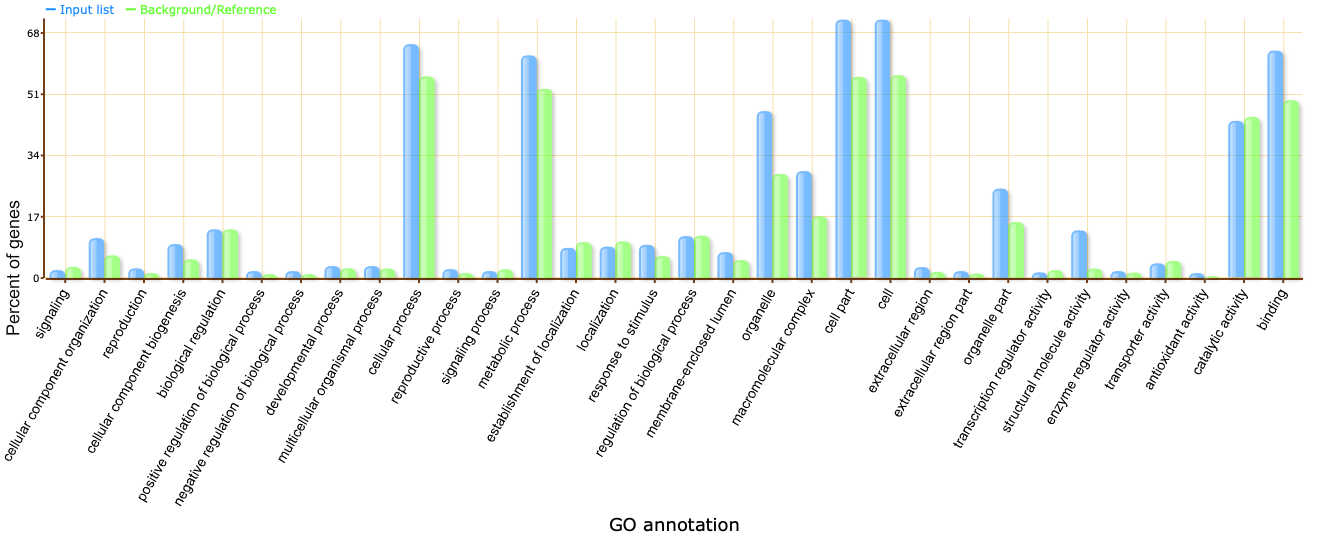

Supplement: Supplementary file 1 [file plants-09-01225-s001.zip › supplementary data/Fig S6.png]

A

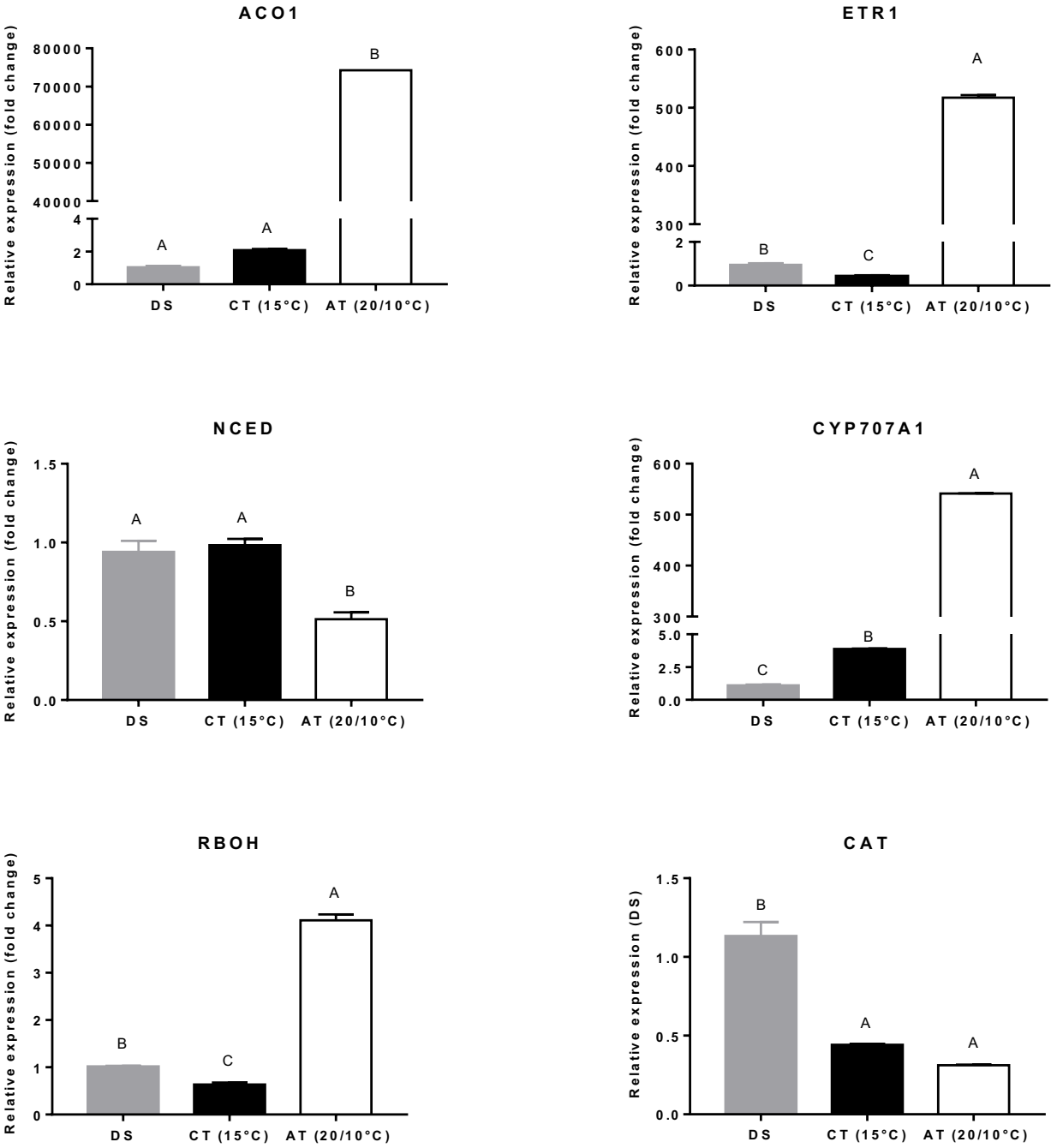

B

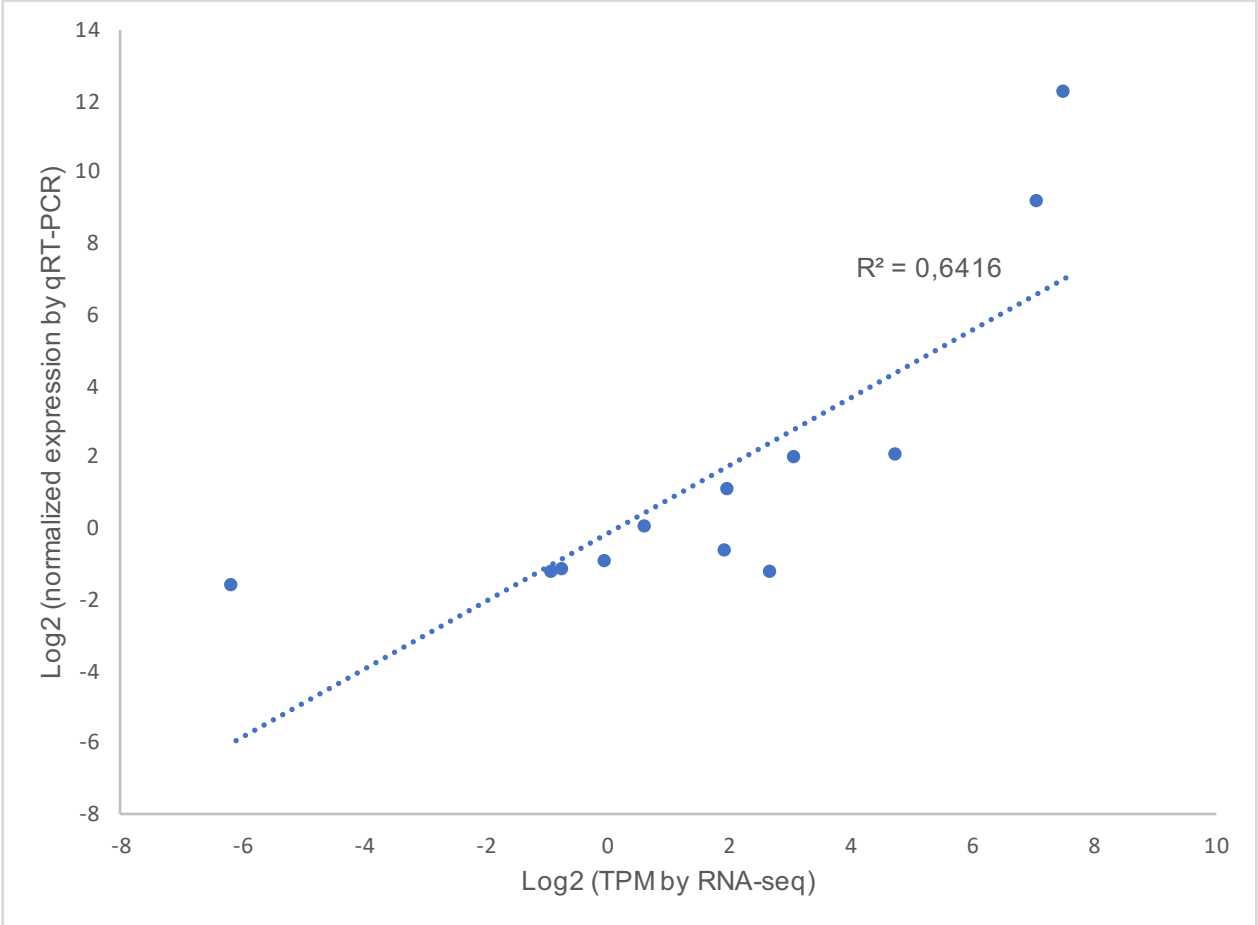

Supplement: Supplementary file 1 [file plants-09-01225-s001.zip › supplementary data/Fig S4.pdf]

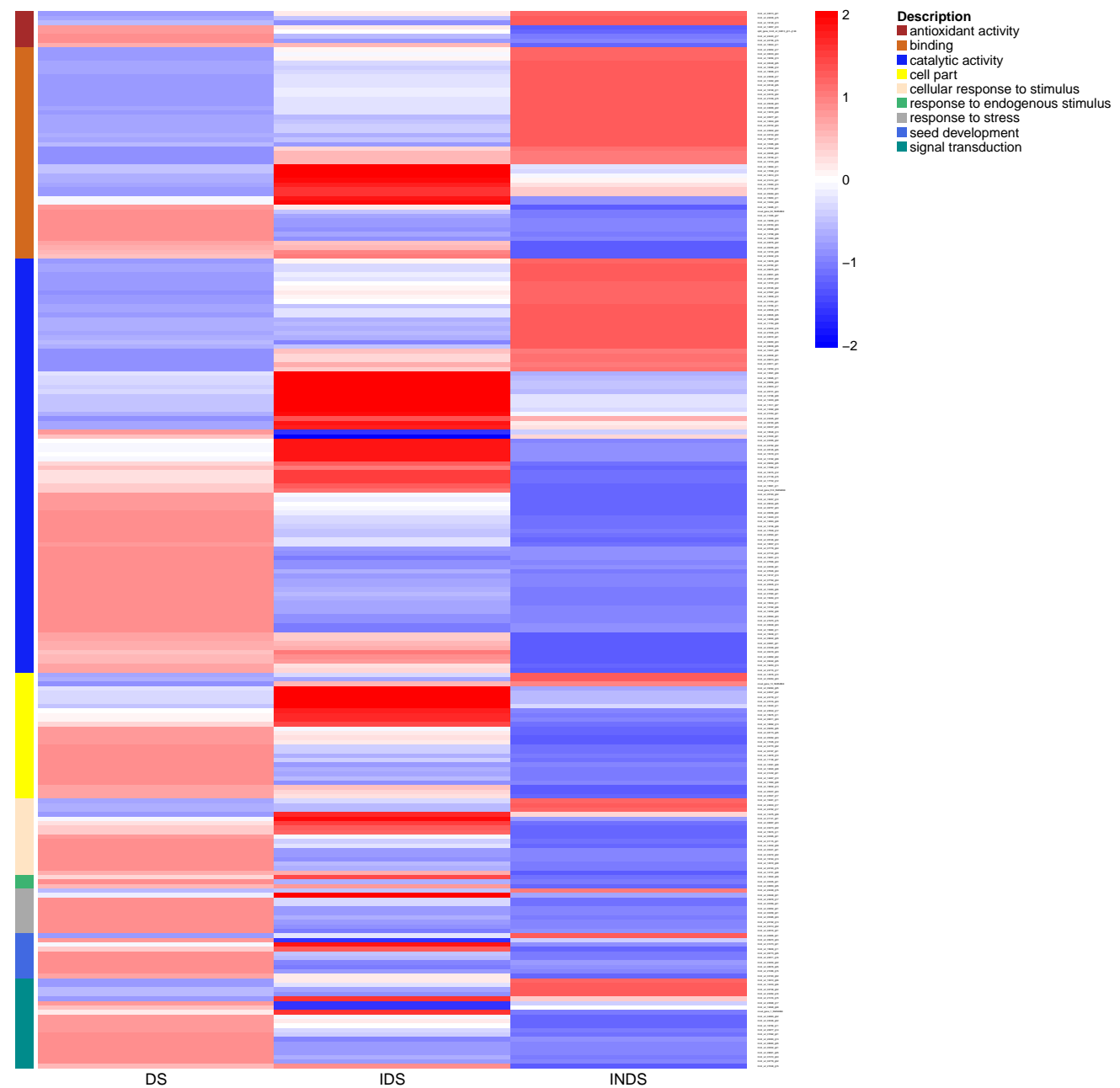

Supplement: Supplementary file 1 [file plants-09-01225-s001.zip › supplementary data/Fig S7.pdf]
